# The Impact of COVID-19 on Waste Infrastructure: Lessons Learned and Opportunities for a Sustainable Future

**DOI:** 10.3390/ijerph20054310

**Published:** 2023-02-28

**Authors:** Poornima A. Jayasinghe, Hamoun Jalilzadeh, Patrick Hettiaratchi

**Affiliations:** Department of Civil Engineering, University of Calgary, Calgary, AB T2N 1N4, Canada

**Keywords:** pandemic, COVID-19, waste management, systematic solutions, waste infrastructure

## Abstract

The onset of the COVID-19 pandemic posed many global challenges, mainly in the healthcare sector; however, the impacts on other vital sectors cannot be overlooked. The waste sector was one of the significantly impacted sectors during the pandemic, as it dramatically changed the dynamics of waste generation. Inadequate waste management practices during COVID-19 shed light on the opportunities for developing systematic, sustainable, and resilient waste infrastructure in the future. This study aimed to exploit the learnings of COVID-19 to identify any potential opportunities in post-pandemic waste infrastructure. A comprehensive review on existing case studies was conducted to understand the waste generation dynamics and the waste management strategies during COVID-19. Infectious medical waste from healthcare facilities had the largest influx of waste compared with non-medical waste from residential and other sectors. This study then identified five key opportunities from a long-term operational perspective: considering healthcare waste sector as a critical area of focus; encouraging the integration and decentralization of waste management facilities; developing systematic and novel approaches and tools for quantifying waste; shifting towards a circular economy approach; and modernizing policies to improve the effectiveness of the post-pandemic waste management infrastructure.

## 1. Introduction

Solid waste generation rates in urban areas around the world are rapidly increasing with the expansion of the population, economic development, changing consumption patterns, and urbanization. More than 2.24 billion tons of solid waste were generated globally in 2020, with a per capita waste footprint of 0.79 kg per person per day, and annual waste generation is expected to increase by 73% from 2020 levels to 3.88 billion tons in 2050, under a business-as-usual scenario [1,2]. If this generated waste is not properly collected, disposed, or treated, it poses numerous threats to the ecosystem and human beings at various scales. 

Managing solid waste in an environmentally friendly manner is essential for the sustainability of urban systems. Waste generation rates and composition differ across countries, income levels, consumption and lifestyle patterns, seasonal variations, and various sectors and infrastructures. Furthermore, financial and political considerations create numerous challenges to the waste management infrastructure of municipalities. In addition to the known challenges, special scenarios, such as natural disasters, generate a large volume of unique waste, typically within a short period of time, posing additional challenges. One such scenario that posed enormous challenges at every level across infrastructure systems, including the waste management infrastructure, was the COVID-19 pandemic. Unlike natural disasters, such as earthquakes and tropical storms, the COVID-19 pandemic disaster did not disappear in a matter of hours or days; it existed over 2 years, shaking every system globally [3]. 

The COVID-19 pandemic had a tremendous impact on the waste infrastructure of municipalities because of an unusual increase in the amount of waste, primarily infectious medical waste [4,5]. The drastic changes in quantity and quality could not be handled by existing (i.e., pre-COVID-19) waste management systems, as they were designed only for moderate variation [6]. While proper waste management is critical for health and safety, it is more critical during times of pandemic, to ensure that potentially infectious materials are disposed of safely to prevent the further spread of the virus [5]. Consequently, every country had to quickly adapt their waste management systems and procedures to effectively manage the demand and to protect essential workers, as well as the general public, from associated threats from the large quantities of infectious waste created. At the same time, the inherent uncertainty, evolving nature, and lack of recent historical precedents to guide informed decision-making further complicated the ability of waste management sectors to properly respond to these new challenges.

One of the well-known phrases in the emergency management infrastructure is that “the onset of a critical incident is the wrong time to exchange business cards”; similarly, “in the pandemic is usually the wrong time to try to install new healthcare waste management systems and practices from scratch”, as highlighted in the report published by the United Nations Environment Programme [3]. Looking at the waste management systems from a resilience perspective is, therefore, very important. Resilience is not a new concept, although the term “resilience” may be less heard in the context of waste management. Resilient waste management systems should be capable of functioning at acceptable levels despite the chronic stresses and acute shocks they experience. To achieve this, the system must be capable of reacting and adapting itself to variations in the availability of infrastructure and, above all, to variations in the amount and types of waste produced [7,8].

The threat of the COVID-19 pandemic is fading; however, the lessons learnt during the pandemic may provide opportunities for developing resilient urban waste management systems, to enable them to handle such scenarios that may arise in the future. Some of the existing literature has discussed the impacts and challenges of COVID-19 on the short-term waste management practices for infectious medical waste [5,6,9,10,11]; however, the lessons that the waste management experts have leant from the pandemic and what strategies have the best potential to be adopted in developing resilient waste management systems in the long-term are not yet well understood and have not received much attention within the research community. This study thus attempted to fill the knowledge gaps by studying the dynamics of waste generation during COVID-19 and identifying long-term opportunities in developing sustainable and resilient waste management strategies. 

A comprehensive literature review on the existing case studies was conducted to observe the waste generation patterns and trends, considering two key waste streams: the infectious medical waste generated from healthcare facilities and the non-medical waste streams generated from residential and other sectors. The review was also extended to study the adopted short-term waste management strategies during COVID-19 and the associated challenges. This study then identified some key long-term opportunities, providing recommendations on potential areas for improvement in the waste management infrastructure. The lessons learnt from the COVID-19 and the potential opportunities are important from a long-term operational perspective, as they could assist urban planners and policymakers to better promote systematic and novel solutions in waste management.

## 2. An Overview of the Waste Generation Dynamics and Associated Waste Management Challenges during the COVID-19 Pandemic

Various existing case studies on the impacts of COVID-19 on waste have shown that the pandemic had major impacts on the waste infrastructure. While the general trends can be seen globally, each municipality had varied experiences and unique challenges based on the level of the impact [12,13,14,15,16,17,18,19,20,21,22,23,24]. Although the data related to the percentage of the change in waste generation before the pandemic and during the pandemic are limited, some researchers have attempted to tabulate the estimated generation of pandemic-related medical waste across different countries [11,23,25,26]. To obtain a holistic understanding on the variations in the waste generation trends across those regions, we will discuss only a few important global statistics and region-specific trends related to the two key waste streams generated during the COVID-19 pandemic: infectious medical waste generated from healthcare-related facilities and non-medical waste generated the from residential and other sectors. Some of the relevant reported statistics on waste generation dynamics are summarized in Table 1, and the subsection below describes the patterns in detail.

Medical waste can be categorized into eight major groups: infectious waste; pathogenic waste; sharps waste; chemical waste; cytotoxic waste; radioactive waste; pharmaceutical waste; and non-hazardous waste [3]. In general, 85% of pre-COVID healthcare waste was reported to be non-hazardous. Undoubtedly, this trend shifted during the COVID-19 pandemic; despite the fact that the percentage of the increase was not reported, all the infected medical waste was considered hazardous [11]. 

The use of a complete PPE set, including gloves, face masks, a head cover, goggles, a surgical gown, and shoe covers, was mandatory for all healthcare and frontline workers to minimize the associated risk. During the early stages of the COVID-19 pandemic, the World Health Organization estimated a global demand of 89 million medical masks, 76 million examination gloves, and 1.6 million goggles per month for healthcare professionals [27]. Another study estimated a global monthly demand of around 129 billion facemasks and 65 billion gloves [28]. The use of PPE during the pandemic not only increases the quantity of medical waste, but also altered the average density of medical waste [15]. Single-use masks, N95 masks, and other disposable PPE, such as surgical gowns and goggles, are primarily made of plastics (>80%), often incorporating polypropylene, polyurethane, or polyacrylonitrile [28]. Therefore, improper disposal of COVID-19 medical waste also contributed to plastic pollution. 

Based on preliminary experience in dealing with COVID-19 patients in China, the Asian Development Bank estimated that 3.4 kg of biomedical waste was produced for every infected patient per day [29]. This estimation was used as a basis in calculating the total waste generation rates in many case studies, given the total number of COVID-19 cases in that region, the rate of hospitalization, and the days of isolation. 

Table 1 summarizes the changes in the waste generation rates during COVID-19 for the medical and non-medical waste streams for selected case studies. 

**Table 1:** Dynamics of waste generation during COVID-19.

Region	Waste Type	Change in Waste Generation During COVID-19	References
Wuhan City, China	Medical Waste	6 times more waste	[15]
Healthcare Facility, Jordan	Medical Waste	10-fold increase	[12]
Malaysia	Medical Waste	27% increase	[18]
India	Medical Waste	10% more waste	[16]
Bangladesh	Medical Waste	80% increase	[22,30]
Tehran, Iran	Medical Waste	17.6–61.8% increase	[17]
Bangkok, Thailand	Medical Waste	5 times more waste	[10,31]
Jakarta, Indonesia	Medical Waste	5 times more waste	[10,31]
Manila, Philippines	Medical Waste	Additional 280 tons/day	[10]
Hanoi, Vietnam	Medical Waste	Additional 160 tons/day	[10]
Kuala Lumpur, Malaysia	Medical Waste	Additional 154 tons/day	[10]
Survey from 23 Countries	Food Waste	43% increase	[32]
Survey from 23 Countries	Plastic Waste	53% increase	[32]
Ontario, Canada	Residential Organic Waste	20% increase	[31]
Ontario, Canada	Residential Garbage	15% increase	[31]
Ontario, Canada	Residential Recyclables	Unchanged	[31]
Ontario, Canada	ICI Waste	Declined, data not reported	[31]
City of New York	Organic Waste	13.3% more waste	[31]
Brazil	Residential Recyclables	25% increase	[21]
Tokyo, Japan	Residential Waste	110% increase	[3]
Tokyo, Japan	ICI Waste	57% decline	[3]

During the height of the outbreak, hazardous medical waste peaked at 240 tons per day in Wuhan City in China, nearly six times more than the pre-pandemic levels. However, they only had an incineration capacity of 49 tons per day of medical waste [15]. A study conducted on the data from a healthcare facility in Jordan estimated a 10-fold increase in the pandemic’s waste generation rates compared with the pre-pandemic rates [12]. Malaysia reported a 27% increase in the generation of healthcare waste attributed to COVID-19-related activities [18]. India produced about 600 tons of biomedical waste daily, which was about 10% more waste as a result of the pandemic [16]. A study conducted in Bangladesh found that there was an 80% increase in medical waste generation within 1 year [22,30]. Medical waste generation in Tehran increased by 17.6–61.8% because of the COVID-19 pandemic [17]. Similarly, cities such as Manila, Jakarta, Kuala Lumpur, Bangkok, and Hanoi experienced up to 150–280 tons/day of additional medical waste [10,31]. The gap between the medical waste generated and the limited existing treatment capacity posed numerous challenges in the waste infrastructure of major cities. 

The lockdowns, stay-at-home orders, travel bans, and worker layoffs caused an increase in non-medical waste generation in most cities. Non-medical waste streams refer to the other general waste streams, including residential waste; industrial, commercial, and institutional (ICI) waste; and construction, renovations, and demolition (CRD) waste. The increases in residential waste were related to the higher level of consumption, including purchasing food in bulk, panic buying of cleaning products and sanitizers, online orders, and the related packaging [5,32]. Both the residential and ICI waste generated during the COVID-19 pandemic included potentially infectious materials, such as masks, gloves, and tissues; however, quantitative information on the infectious vs. non-infectious categories in the residential and ICI waste streams are not available. 

A study conducted by Filho et al. [32] showed increases of 43% in food waste and of 53% in plastic packaging compared with pre-pandemic levels of waste generation. On the other hand, there seemed to be variation relative to the waste generation from different subsectors, and the potential tonnage burden shifted between the ICI and residential sectors. The trends in ICI and CRD waste indicated that the collection tonnage of ICI and CRD waste generally deceased due to diminished activities. According to Roy et al. [31], the residential waste generation rate significantly increased in Ontario, Canada, by 15% for garbage and 20% for organic waste, while the recyclables remained unchanged, but a decline in ICI waste was observed. During the COVID-19 pandemic, the city of New York generated 13.3% more organic waste [31]. In Brazil, the recyclable residential waste material in cities increased by nearly 25% [21]. Similarly, a 110% increase in residential waste over pre-COVID levels was reported in Tokyo, along with a 57% decline in ICI waste generation caused by business closures and lockdown protocols [3]. While the majority showed a similar increasing trend of residential waste, some cities also showed contradictory behavior, with a decreasing trend during different waves of the pandemic, possibly related to the variation in consumption patterns and the associated socioeconomic stresses [11]. 

The statistics from various case studies globally showed that the infectious medical waste generated from healthcare facilities had the largest influx in terms of the changes in waste generation dynamics compared with the residential and other sectors. Waste streams from the residential and other sectors included more potentially infectious materials than before; however, such information is limited in the literature, because of the complexities of collecting such data. The associated challenges in the waste infrastructure during the COVID-19 pandemic included the lack of proper medical waste management systems in place to handle the sudden surge in infectious biomedical waste; the increased the demand for single-use plastic, because of the required use of personal protective equipment (PPE) and other healthcare items; decreases in recycling activities and diversion to landfills and illegal dump sites; increases in mixed waste, including infectious waste with low levels of segregation at the source; increases in residential waste volumes overwhelming the existing municipal solid waste management systems; and decreases in ICI waste caused by the slowdown in the economic sector. To accommodate these variations and to protect the workforce and public from infection, waste management systems and procedures needed quick adaptations. 

**Figure 1:**
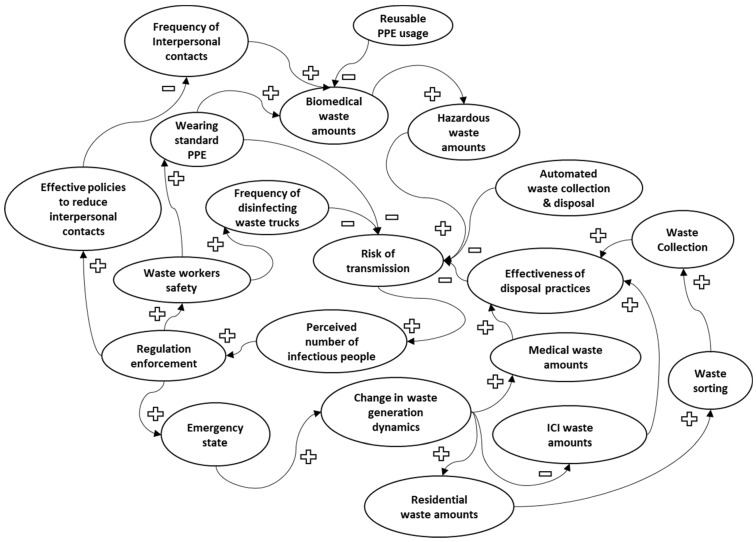
Causal loop diagram for COVID-19-related waste generation.

To summarize the learnings from the case studies and to grasp the complexity of the impacts of COVID-19 on waste infrastructure, we developed a causal loop diagram (CLD), as shown in Figure 1. The CLD consists of a few basic elements: the variables; the links between them; the signs on the links; and the loops to show how the variables are interconnected. The CLD in Figure 1 identified minimizing the risk of transmission as one of the key goals when dealing with the waste during the pandemic. As mentioned previously, the general trends of the shifts in waste indicated increases in residential waste and decreases in ICI waste. However, the different increases and decreases in quantities between different subsectors would have mostly offset themselves in terms of the total quantity. As seen from the case studies, some cities were able to carry out their business-as-usual routine once they overcame the initial challenges associated with the capacity of the existing waste treatment facilities. Therefore, the effectiveness of treatment/disposal practices also played a key role in minimizing the risk of transmission. Finding ways to manipulate the system to produce minimal amounts of waste, promote reuse at higher levels, and design automated waste collection systems could aid in minimizing the risk of transmission as well, and could prove to be effective when dealing with potentially similar conditions in the future. 

## 3. Key Lessons Learnt and Opportunities

As discussed previously, the COVID-19 pandemic has had a profound effect, not only on human health but also on the environment, creating many challenges across every municipal infrastructure at different scales. While it was important for the authorities around the world to treat the public health sector as the main focal point during the pandemic, other vital sectors cannot be overlooked. Although it is very critical, one of these under-noticed sectors, which eventually needed attention to prevent massive social and environmental disruption, was the waste infrastructure. A lack of appropriate elements in the waste infrastructure system, such as safe waste disposal systems to tolerate the sudden surge of medical waste and plastic-based waste streams, comprehensive and systematic tools, alternative and adaptable technologies, and policies became the key challenges. If taken seriously and positively, these challenges also create opportunities for improvements and innovations. The subsections below elaborate on the key challenges, identifies the lessons leant, and provides some potential opportunities for developing a resilient and sustainable waste infrastructure in the long-term.

### 3.1. Healthcare Waste Management Systems

As the number of hospitalizations surged, the amount of waste from healthcare facilities around the world grew beyond the handling capabilities of waste management systems. The ability of each country or urban system to manage the increase in the quantities of medical waste depends on several factors, such as the making and enforcement of policy; existing collection, transportation, disposal, and treatment methods and facilities; the existing procedures and capacities to treat medical waste; and the allocated medical waste management budgets [5,9]. Appropriate identification, collection, separation, storage, transportation, disposal, and treatment must become part of the effective management of medical waste. Subsequently, productive medical waste administration is another area needing special attention; this includes understanding the specified standards, disinfection guidelines, protection of personnel and the associated risks, training, and aspects of monitoring [33,34].

Healthcare waste management has been always a challenge. Many countries, especially developing nations, lack proper handling and disposal procedures for medical waste, leading to adverse environmental impacts, such as groundwater and air contamination, and the potential for viral outbreaks. According to the World Health Organization [35], 30% of healthcare facilities (60% in the least developed countries) are not equipped to handle the existing waste loads, let alone the additional load of COVID-19. Taken positively, the pandemic has forced these countries to rectify these neglected aspects and consider healthcare waste management as a critical area of importance.

The financial constraints to applying proper collection, transportation, disposal, and treatment processes, coupled with a general lack of education and guidelines on the proper handling of medical waste, were identified as some of the key challenges in implementing safe waste management practices [25,36]. In response to the COVID-19 outbreak, many organizations, including the United Nations Environment Programme (UNEP), the World Health Organization (WHO), the European Union (EU), and the European Centre for Disease Prevention and Control (ECDC), published revised management guidelines for infectious waste, putting more weight on the COVID-19-related infectious medical and household waste streams [9,14,37,38,39,40]. In addition, a set of best practices for the safe handling of medical waste have been proposed by different researchers, as this area has received more significant interest within the research community than ever before [4,6,8,9,11,16]. Now is an appropriate time to look into the medical waste sector closely and integrate these guidelines and best practices, if they have not yet been integrated into the existing systems, while also considering medical waste management as one of the critical areas of the waste infrastructure [14].

The inability of the existing treatment facilities to meet the variations in the extra waste generated by healthcare centers was another area of concern during the COVID-19 pandemic. The capacity constraints of treatment facilities in some regions resulted in the illegal dumping of waste in suburban areas and uncontrolled burning. Considering the long-term environmental and health consequences, the improper waste disposal perpetuated by the pandemic could lead to environmental devastation in the post-pandemic world [9,25]. It is also important to note that the strategies used to manage pandemic-related waste in all regions were not similar: some of the commonly used practices to handle the extra quantities of waste included safe temporary storage and temporary treatment, providing alternative systems such as mobile incineration or autoclaving, using cement kilns and other industrial furnaces as alternative treatment facilities, and using alternative technologies [6].

Two of the most common management techniques used in the medical waste sector are incineration and safe disposal in landfills [14,41]. Uncontrolled incineration or incinerating unsuitable materials may release toxic air pollutants and other toxic compounds, including ash residues [36]. For landfill disposal, the solid waste must be rendered non-pathogenic by onsite chemical or thermal sanitization processes before being sent to the landfill. The disposal of untreated healthcare waste in poorly constructed landfills can lead to contamination of the water, soil, and air [41]. From a treatment point of view, integrating alternative technologies other than having to rely on one treatment method may have been a better approach to mitigate the extra demand. Some of the viable alternative technologies include autoclaving, microwaving, gas sterilization, irradiation, thermal inactivation, chemical disinfection, and hydrothermal carbonization [9]. However, adopting any of these technologies would depend on many conditions, including their affordability, adaptability, capability, technological maturity, and economic and environmental concerns.

### 3.2. Integrated and Decentralized Waste Management

The COVID-19 pandemic was far more than a global health crisis: it affected the overall society, economy, and every infrastructure system, adding both chronic and acute shocks. Returning to normal is challenging; developing a long-term, systematic, and integrated plan for the solid waste infrastructure is necessary.

The limited resources and technology options, and the limited adaptability and capability of existing waste generation, collection, transportation, and disposal systems for managing the increased volume of healthcare waste were identified as some of the key challenges in addressing the COVID-19-related waste [16,42]. One activity or strategy alone is insufficient to effectively mitigate the risks associated with waste. Using a combination of many activities represented in the integrated solid waste management (ISWM) model could be the best approach. The ISWM represents a strategic approach to the sustainable management of solid waste, covering all the sources, activities, and aspects associated with the management of waste. In this context, the ISWM hierarchy refers to the five R’s of reduction of waste at the source, reuse of products, recycling materials, recovery of energy, and residual management, and could be considered an effective tool [43]. This approach promotes a less complicated proactive waste management approach, where anticipating the issues and preventing them from happening by producing less waste or zero waste, as opposed to the more complicated reactive approach of reacting to issues as they arise by the final treatment of waste.

The waste infrastructure is highly interconnected with other critical infrastructures of an urban system, as they function as a “system of systems”, exhibiting simple to complex interdependencies that can leave critical functions vulnerable to cascading failures [44]. In other words, a waste management system is organized in the form of networks, as it includes support networks (roads, buildings such as incineration facilities, storage centers, utilities, etc.) comprising several points or nodes and a network [7]. This relationship is bidirectional: every infrastructure system in an urban system generates some kind of waste, while the waste infrastructure also depends on all the other infrastructures. Waste treatment facilities themselves are physical buildings; landfills compete with other infrastructures for space; waste collection relies on trucks and transportation; and sorting facilities need energy. It is important to look at these interdependencies positively, without eliminating the impacts, as they provide opportunities for enhancing resilience and sustainability [45]. As opposed to the traditional silo-based approach of analyzing infrastructures in an isolated manner, an integrated infrastructure system approach offers considerable benefits by enhancing system-wide efficiencies and resilience; minimizing service disruptions and rehabilitation work and costs; promoting sustainability goals including resource minimization and proper allocation, and looking into the lifecycle of individual components; and identifying and managing short- and long-term impacts, as well as involving many other environmental and economic benefits [46,47,48,49].

The integration of infrastructures can take multiple forms. One of the attractive dimensions is merging integration and decentralization, where integration happens in a de-centralized way other than limiting the integration only in a centralized location [46]. The surge in healthcare waste mostly suffocated the existing waste management systems during COVID-19; however, the expansion of healthcare facilities and the construction of new facilities, such as hospitals, isolation wards, temporary quarantine camps, and testing centers, posed extra challenges in terms of following the protocols related to waste segregation, storage, transportation, and disposal [9,25]. Apart from the capacity constraints of central waste management facilities, the absence of in-situ or nearby waste treatment facilities increased the transportation of bulk amounts of infectious waste over a long distance, leading to further contamination and impacting the safety of the workers. This highlights the importance of integrating decentralized and onsite waste management facilities into the waste infrastructure [25]. Some simple practices include in-house waste segregation, in-situ biodigesters, incineration units, and backyard composting. Decentralized systems with active citizen participation and following the 5R principles in ISWM may help urban centers become less dependent on the central waste management facilities [50].

### 3.3. Quantity and Quality of Waste, System Thinking, and Novel Tools

During the COVID-19 pandemic, daily updates and global statistics were available related to public health and environmental concerns, such as the number of cases and deaths, and real-time air pollution monitoring data. However, widespread access to waste generation, composition, and recycling data were not available in a similar format. Although challenging, it is important to gather reliable, real-time information about the quantity and quality, including the amount and type, of waste generated, the amount already recycled, and the amounts to be disposed of. This information would be useful for the waste management industry to identify the key issues promptly, as well as to identify and prioritize the mitigation measures urgently, especially in emergency situations [51,52]. On the other hand, public awareness of these existing challenges might have had some positive impacts on the waste infrastructure through changes in behavioral and consumption patterns.

As evident from the case studies discussed previously, pandemic-related measures across different sectors had different impacts on sectoral waste generation trends. Working from home increased residential waste while reducing the waste generated by businesses and schools. In order to understand and accurately evaluate the quantity (i.e., waste generation volumes or rates by a sector, and waste composition) and quality (i.e., infectious vs. non-infectious) of the waste, a comprehensive set of models, such as system dynamic models and input–output models, are needed. For example, a systems thinking approach, coupled with causal loop diagrams, would be useful for better understanding these trends and also the interdependencies among the waste sectors; one such diagram is shown in Figure 1.

Another tool that can be applied to quantify the mass and energy balances of the inputs, outputs, production, consumption, and waste of an urban system is the urban metabolism approach [46]. Quantitative and qualitative models and tools should be simple enough to use and validate under different scenarios but, at the same time, they should incorporate all the complex and interdependent elements to better represent the system.

However, these models and tools cannot function in the absence of reliable data. Adjusting existing waste management systems, improving efficiencies, and integrating new systems also require consistent and reliable data. While data scarcity is also a critical issue to investigate, comprehensive models and novel tools could be used to predict and estimate the data, such as the generation rates and composition of waste [53]. The lack of such tools and models, therefore, imposes an extra burden in situations such as the COVID-19 pandemic.

As opposed to the application of conventional tools, there are opportunities to introduce novel and robust approaches, such as using artificial intelligence (AI)-enhanced tools and techniques in waste infrastructure, especially for accurately forecasting the trends of waste. Various AI-coupled models, such as artificial neural networks (ANN), support vector machine (SVM), decision trees (DT), and genetic algorithms (GA) techniques, were shown to have had a significant impact on the prediction, classification, collection, and transportation of waste, and also in the modelling and optimization of waste treatment [53,54,55,56,57]. Accurate forecasts of waste trends using AI-enhanced tools would play a key role in decision making in waste demand management andoptimization of waste collection routes and frequency, providing many environmental and economic benefits. Some examples of applying AI-based techniques in waste management include smart recycling bins, automated sorting systems, waste collection robots, and autonomous waste collection trucks [57,58]. These techniques are immune to infection, and they avoid human contact and contamination, which can play a significant role in the battle against infectious diseases such as COVID-19.

Furthermore, enhanced healthcare waste prediction models and tools, coupled with the associated economic indicators and social elements, such as a non-linear, multi-level regression model, would be useful in planning, designing, budget allocation and optimization, and enhancing sustainability in the healthcare waste management sector [59,60].

### 3.4. Product Design and the Circular Economy Framework

During the COVID-19 pandemic, the demand for single-use plastic increased significantly because of the required use of PPE, sanitary, and other healthcare items, and packaging. This situation also encountered supply chain issues, especially when these systems relied on disposables. Despite the fact that plastics played an essential role in surviving the pandemic, the ever-increasing mass of unmanaged plastic waste is causing a global ecological disaster, as they could lead to microplastic pollution and its potential implications for the environment and human health, considering short- and long-term scenarios [52]. Plastic waste management has always been a challenge. Most of the plastic waste accumulated during the COVID-19 pandemic was landfilled or incinerated, and a minor fraction was recycled, mainly because potentially contaminated plastics were restricted at recycling centers [11,60]. Plastic waste does not belong in landfills, as it may not decompose; incineration could also lead to air pollution issues.

Although it would be a challenging shift, immediate action is therefore required in shifting towards sustainable production, sustainable consumptions, and exploring recycling and alternative treatment technologies in plastic waste management. In this context, shifting towards the circular economy approach has received a significant attention over recent years, as opposed to the traditional and unsustainable linear economy approach [61,62,63,64,65,66,67]. The circular economy promotes cyclical flows of resources in the production–consumption system within a closed loop, looking throughout the system’s lifecycle to minimize the consumption of natural resources and energy, achieve zero waste goals, and mitigate the associated environmental impacts, while also providing opportunities for identifying the best practices and thus, moving towards sustainability.

Eco-designs and biodegradable products play a vital role in the circular economy, as it also considers the “cradle-to-cradle” approach as a framework. Looking into all the lifecycle stages during product design encourages best practice, such as the utilization of secondary and recycled resources as raw materials, developing eco-designs and utilizing biodegradable materials in manufacturing, and increasing recycling and energy recovery from waste. However, full techno-economic and environmental footprint assessments for industrial-scale applications are needed for integrating products such as bioplastics in the market [61]. The evidence from initiatives during the COVID-19 pandemic included producing disinfectants from residue products and making re-usable cloth masks. These practices not only used the locally available resources to produce sustainable products, but also supported the supply chain without having to depend on imported items and provided economic savings for consumers [65]. Integrating the circular economy in the healthcare sector is therefore imperative, not only to be sustainable and resilient in the long term, but also to minimize the dependency on imports in the critical healthcare value chain.

Researchers in the past have also suggested that implementing systematic and greenification strategies, such as reductions in medical waste; the adoption of green energy sources, the reuse/recycling, repair, and refurbishment of medical products, the efficient usage of resources, and sustainable procurement as key factors in elevating the sustainability of the healthcare sector [59,68,69].

Furthermore, along with preventing, reducing, and recycling waste, there is also a need to develop new technological approaches, such as integrated mechanical and chemical recycling processes, to improve end-of-pipe plastic treatment, despite it being the least preferred option in the waste management hierarchy.

### 3.5. Implications for Policy, Protocols, and Guidelines

As highlighted by some researchers, the inadequacy of existing policies, protocols, and guidelines in the waste infrastructure restricted authorities from taking immediate action in the collection, classification, transportation, and disposal of waste, particularly at the beginning of the crisis [9,14]. Some of the identified issues include the following: illegal waste disposal in some places added an additional risk to the community; illegal waste pickers were vulnerable; and waste operators had trouble continuing manual collection and sorting practices. Integrating proper waste disposal practices and providing the people with protective logistics is, therefore, imperative.

Similar to other measures discussed previously, it is appropriate to enforce global waste management and local authorities to critically revisit and improve the existing policies, protocols, guidelines, and best practices as needed to be able to improve the effectiveness of the post-pandemic waste infrastructure. Instead of approaching emergency management from a business-as-usual approach, there needs to be a thorough analysis of the short-term, long-term, and absolute worst-case scenarios when developing policies to improve waste management’s resilience and efficiency. Some of the examples include revisiting international and country-specific guidelines on biomedical waste management; revising emergency preparedness measures in the waste infrastructure; allocating cost-effective and temporary facilities that can be utilized to continue waste management operations; revisiting local collection, handling protocols, and the protection of sanitation workers; forbidding manual waste sorting; enhancing the policy and regulatory frameworks set in place to minimize the use of single-use plastics; enhancing funding for existing waste management initiatives; providing consumers with economic incentives to minimize waste generation; extending public–private and national–international partnerships; promoting extended producer responsibility (EPR) and collaborations in decision-making; incorporating trash metering and a usage pricing model for waste disposal; and increasing public visibility and awareness.

**Figure 2:**
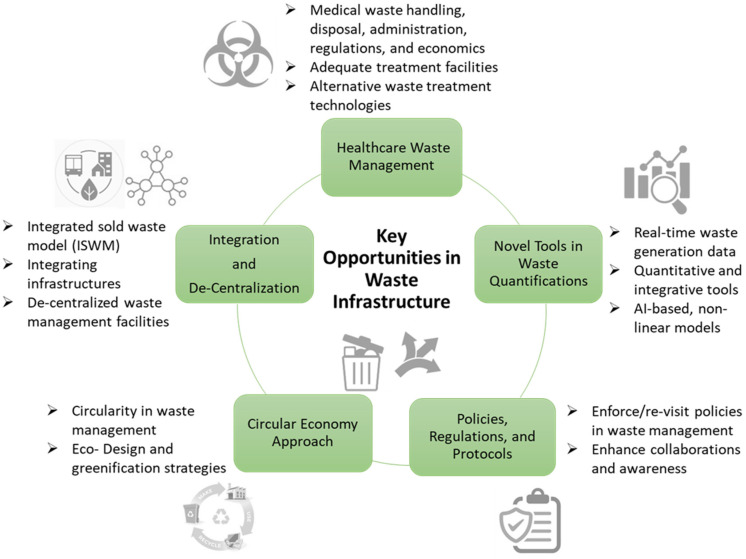
Opportunities in the post-pandemic waste infrastructure.

Figure 2 summarizes all the key the potential opportunities described above. In addition to all the techno-economical and environmental dimensions discussed, there also need to be social cooperation and behavioral changes to uphold the appropriate waste management practices. One of the key points is that waste management is not only the responsibility of the authority or the waste managers but is a collective effort: every citizen needs to be vigilant and responsible for their individual actions and consumption patterns.

## 4. Conclusions

The COVID-19 pandemic impacted the waste infrastructure significantly, as it changed the dynamics of waste generation beyond the capabilities of the existing waste management systems. The infectious medical waste generated from healthcare facilities across the world increased extensively, mainly because of the use of mandatory PPE during the COVID-19 pandemic. According to the review conducted here, the residential waste streams showed an increasing trend, which was somewhat counterbalanced by reductions in other waste streams, such as the ICI waste stream. While it is understood that the nature and the impacts of the COVID-19 pandemic are different from those any of the other recent crises, it is now time to react by taking the learnings as opportunities to identify the inadequacies of the waste management infrastructure and take the necessary measures. Five key long-term opportunities were proposed in this study to develop a sustainable and resilient waste management systems, as follows: managing healthcare waste appropriately considering it as a critical area of focus; encouraging the integration and decentralization of waste management facilities; developing systematic and novel approaches and tools for waste quantification; shifting towards a circular economy approach; and modernizing policies to improve the effectiveness of the post-pandemic waste management infrastructure. These takeaways would be useful in planning, designing, and implementing integrated waste management strategies in the long run, moving towards urban sustainability and resiliency; however, developing such an integrative waste management system is very challenging. It requires multi-disciplinary knowledge and expertise, policies, financing, social acceptance, and behavioral changes.

## Data Availability

Not applicable.
